# A numerical algorithm with preference statements to evaluate the performance of scientists

**DOI:** 10.1007/s11192-014-1521-2

**Published:** 2015-01-22

**Authors:** Martin Ricker

**Affiliations:** Instituto de Biología, Universidad Nacional Autónoma de México (UNAM), Ciudad Universitaria, 04510 México, D.F. Mexico

**Keywords:** Academic evaluation, Evaluation committee, Scientists’ value, Sistema Nacional de Investigadores (Mexico), UNAM’s PRIDE (Mexico)

## Abstract

Academic evaluation committees have been increasingly receptive for using the number of published indexed articles, as well as citations, to evaluate the performance of scientists. It is, however, impossible to develop a stand-alone, objective numerical algorithm for the evaluation of academic activities, because any evaluation necessarily includes subjective preference statements. In a market, the market prices represent preference statements, but scientists work largely in a non-market context. I propose a numerical algorithm that serves to determine the distribution of reward money in Mexico’s evaluation system, which uses relative prices of scientific goods and services as input. The relative prices would be determined by an evaluation committee. In this way, large evaluation systems (like Mexico’s *Sistema Nacional de Investigadores*) could work semi-automatically, but not arbitrarily or superficially, to determine quantitatively the academic performance of scientists every few years. Data of 73 scientists from the Biology Institute of Mexico’s National University are analyzed, and it is shown that the reward assignation and academic priorities depend heavily on those preferences. A maximum number of products or activities to be evaluated is recommended, to encourage quality over quantity.

## Introduction

In Mexico, a traditional stepwise promotion system for scientists is complemented with a periodic performance evaluation system that contributes additional income to scientists’ salaries. The traditional levels at Mexico’s National University (Universidad Nacional Autónoma de México or UNAM) for scientists are *Investigador Asociado* (“associate scientist”) and *Investigador Titular* (“full scientist”), each with levels “A”, “B”, and “C”. In addition to the basic salary according to those levels, there are two reward payments based upon performance. For that reason, scientists are evaluated not only for promotion to a higher category in the traditional sense, but also every few years according to their productivity during the last period. At the UNAM, the reward system is called *Programa de Primas al Desempeño del Personal Académico de Tiempo Completo* or PRIDE (“Bonus Program for the Performance of Full-time Academic Employees”). According to the level, the reward money is paid together with the salary, as a percentage of the base salary: an additional 45 % for level “A”, 65 % for “B”, “85 % for “C”, and 105 % for “D”. Similar reward programs exist also at other Mexican academic institutions.

The second system is the nationwide *Sistema Nacional de Investigadores* or SNI (“National System of Scientists” or “Researchers”). The reward payment is monthly, in terms of minimum wages: six minimum wages for level “I”, eight for “II”, and 14 for “III”. Given the official 2014 daily minimum wage of 67.20 Mexican Pesos, an exchange rate of 12.5 pesos per US dollar, and the usage of 30 days per month, the monthly reward payments are US$969 for level “I” and US$2,261 for level “III”. Evaluation for promotion and assignation of the reward level in each of the two systems are carried out independently by different evaluation committees.

The SNI was established in 1984, while UNAM’s PRIDE originated under a distinct name in 1990, and as PRIDE in 1994. Both programs resulted from an effort in academia to increase the income of scientists and avoid Mexican “brain drain” (in 1982 was a Mexican debt crisis with severe economic consequences). The Mexican Department of the Treasury (Secretaría de Hacienda y Crédito Público) insisted that such increases should be performance-based, as well as without long-term obligations, such as higher pensions.

The nationwide SNI has strongly determined the focus of scientists to publish international articles, written in English and indexed in the *Journal Citation Reports*, elaborated and sold by the Thomson Reuters company (Ricker et al. 2009). It has been successful in leading Mexican scientists to increase exponentially the number of international indexed articles over the last decades in all scientific disciplines (González-Brambila and Veloso 2007: Figure 1; Luna-Morales 2012). Though application to the SNI is voluntary, and the evaluation procedure has generated discussion (if not polemics; e.g., Ricker et al. 2010), being member of the SNI continues to be considered a standard for prestige and recognition for promotion, funding, and rewards. Furthermore, the SNI has also provided the lead for other reward programs, such as the PRIDE.

Both systems in principle contemplate a wide array of possible products and activities, though quantitative criteria are not specific, i.e., evaluation committees have a wide margin to decide according to own criteria. The SNI has received a negative ruling for its operation in 2009 by the *Auditoría Superior de la Federación*, the institution in charge of auditing government programs for Mexico’s Congress (*Auditoría de Desempeño 09*-*1*-*3890X*-*07*-*0187*). The report criticizes (on its pages 12–13) that the products generated by Mexican scientists in the system (15,565 in 2009) during the period 1984–2009 consisted overwhelmingly of publications of articles, books, and book chapters, but very few patents and no technological developments, innovations, or transfers. This, they state on page 16, is in contrast to the program’s objectives that Mexican scientists should be stimulated to contribute to culture, productivity, competitiveness, and social well-being. Furthermore, they ask that evaluation committees should state in their reports how different products were weighted for the evaluation (page 15). The approach proposed here addresses specifically this last point.

## Searching for methods of semi-automated evaluation of scientists

The experience is that both reward systems, the PRIDE and the SNI, struggle to evaluate many scientists in academic evaluation committees, without a framework how to weigh different product categories relative to each other, e.g., publishing a book against publishing less articles. Moreover, if already the regular peer review of articles may cause discussion and disagreement, because of different opinions and views among peers, a qualitative assessment of individual products by evaluation committees, where the presence of true peers is the exception, seems impossible. The problem to adequately evaluate scientists is by no means unique to Mexico, but considered difficult worldwide (Korhonen et al. 2001: 121). The simplest way of evaluating a scientist in this context consists in counting the number of his or her articles published in scientific journals, compare that number against some institutional average, and take other products and activities only marginally into account. The contributions are considered qualitatively only insofar in that the articles were published in journals indexed in the *Journal Citation Reports*. Such an approach has become widespread also in other countries (e.g., García-Aracil et al. 2006, in Spain).

Some academic evaluators favor a more bibliometric evaluation, considering citation counts and bibliometric indexes. Carrying out such a sequence of computational steps that transforms curricular input into evaluation output, represents an algorithm (Cormen et al. 2009: 5). If carried out quantitatively, it represents a numerical algorithm. Even though many people are aware that numerical algorithms are problematic as stand-alone tools for academic evaluation (Ricker et al. 2009; Alberts 2013; Gagolewski 2013; Waltman et al. 2013), in practice evaluators have been increasingly receptive for using them as “almost” stand-alone tools. The reason is that this approach has a number of advantages:

- The evaluation can be semi-automatic, and thus is a quick process that can be carried out in a centralized way repeatedly every few years. In that sense, it is also cheaper and more efficient, because scientists can focus on academic activities other than evaluating their colleagues.
- It is generally considered objective and fair in the sense that the same criteria are applied equally to all. Popper (1959: 44, citing Immanuel Kant) writes that “a justification is objective if in principle it can be tested and understood by anybody”.
- If well implemented, the outcome of evaluations is transparent and largely predictable in advance.

On the other hand, there are important limitations to using numerical algorithms for academic evaluation:

1. Evaluation criteria (preferences) need to be established quantitatively for each academic product category.
2. There is no generally accepted method how to measure creative and innovative scientific work. There is not even a universal definition of what constitutes creativity (Batey 2012).
3. Numerical algorithms tend to be inflexible to account for particular situations that merit exception. Consequently, an algorithmic, quantitative evaluation can interfere negatively in a profession where academic freedom, independence, and experimenting under uncertainty are important for constructive results.
4. There tend to be negative side effects for academic quality, because scientists start “serving the algorithm” rather than science and society. Examples are repetitive publishing, in a time when access to articles is getting ever easier via the Internet, product fragmentation, even when an integrated approach would be more useful, or premature termination of students’ theses, with the objective to get the reward rather than to teach students.

Here, I first analyze as a case study the production and activities during 3 years of 73 scientists at UNAM’s Instituto de Biología (Biology Institute) in Mexico City. Then I propose a semi-automated algorithm that addresses largely the limitations 1–4, and test the approach with the data from the Biology Institute.

## Case study of UNAM’s Biology Institute

At the end of each year, all academic employees of UNAM’s Biology Institute submit an individual report that specifies products, activities, and services, finished during the year. The director presents subsequently an integrated report about the activities of the whole institute during the year, which is made public in the Internet. I used the data for the 3-year period 2010–2012. At the end of 2012, the Biology Institute had 73 scientists and 84 academic assistants (“Técnicos Académicos”). Since the evaluation of academic assistants is different from scientists, only the group of scientists was considered in this case study.

Products and activities considered here are scientific articles (subdivided into “indexed” in Thomson Reuter’s *Journal Citation Reports* or “non-indexed”, and “first author” or “coauthor”), book chapters (“first author” or “coauthor”), books (“first author” or “coauthor”), major advisor of theses finished by students (“undergraduate”, “master’s thesis”, “doctoral thesis”), courses taught as major professor (“undergraduate”, “master’s level”, and “doctoral level”), and technical reports of applied projects for industry, government, or other institutions (“first author” or “coauthor”). For simplification, I excluded some categories, such as work as book editor, peer review of manuscripts, congress reports, or non-scientific articles (e.g., in newspapers). This does not mean that such products should or could not be taken into account.

A matrix in *Excel* was generated, with 73 rows for the scientists and the columns with the 16 products or activities per scientist during 2010–2012. In addition, for articles, book chapters, books, and technical reports as first author, the total number of pages was counted for each scientist. The statistical and mathematical analyses were carried out with *Mathematica* 10.

Table 1 presents the summary statistics for the whole data set. For each variable, the number of data points, the minimum, maximum, median, mean, and the standard deviation are given. Overall, the median number of products per scientist over the 3 years was 14, and the mean 15.0. The range was from 1 to 44 products per scientist. The sum of the number of pages completed as first author was in the wide range from 0 to 850, the median being 26 pages per scientist. There is no significant statistical relationship that would indicate on average a lower number of pages for indexed articles of a scientist with a higher number of published articles as first author. One has to keep in mind, nevertheless, that comparing simply the sum of products among scientists may be unfair, because a major international scientific book can represent a hugely higher value than, e.g., several coauthored articles.

**Table 1:** Statistical summary of per-scientist output from 73 scientists of UNAM’s Biology Institute for the period 2010–2012 (3 years)

	Number	Minimum–maximum	Median	Mean	SD
Number of scientific articles					
Indexed articles (as first author)	73	0–11	1	1.5	1.9
Indexed articles (as coauthor)	73	0–29	4	5.0	4.9
Non-indexed articles (as first author)	73	0–8	0	0.4	1.2
Non-indexed articles (as coauthor)	73	0–5	0	0.8	1.2
Total of articles	73	0–33	6	7.7	5.9
Number of book chapters and books					
Book chapters (as first author)	73	0–7	1	1.2	1.5
Book chapters (as coauthor)	73	0–6	0	0.8	1.2
Books (as first author)	73	0–2	0	0.1	0.4
Books (as coauthor)	73	0–7	0	0.2	0.9
Number of theses as major advisor					
Undergraduate theses	73	0–6	1	1.0	1.3
Master’s theses	73	0–5	1	0.9	1.1
Doctoral theses	73	0–2	0	0.4	0.7
Number of courses taught					
Undergraduate courses	73	0–9	0	1.1	2.2
Courses for master’s students	73	0–5	0	0.7	1.1
Courses for doctoral students	73	0–6	0	0.5	1.2
Number of technical reports					
As first author	73	0–7	0	0.2	0.9
As coauthor	73	0–8	0	0.2	1.0
Pages (as first author)					
Indexed articles	48	2–90	18	25.1	20.8
Non-indexed articles	15	1–45	11	15.6	12.1
Book chapters	41	1–238	19	34.2	46.3
Books	8	38–782	223	305.5	287.3
Technical reports	6	6–683	40.5	151.7	264.5
Number of all products	73	1–44	14	15.0	8.9
Sum of pages (as first author)	73	0–850	26	84.8	166.9

**Fig. 1:**
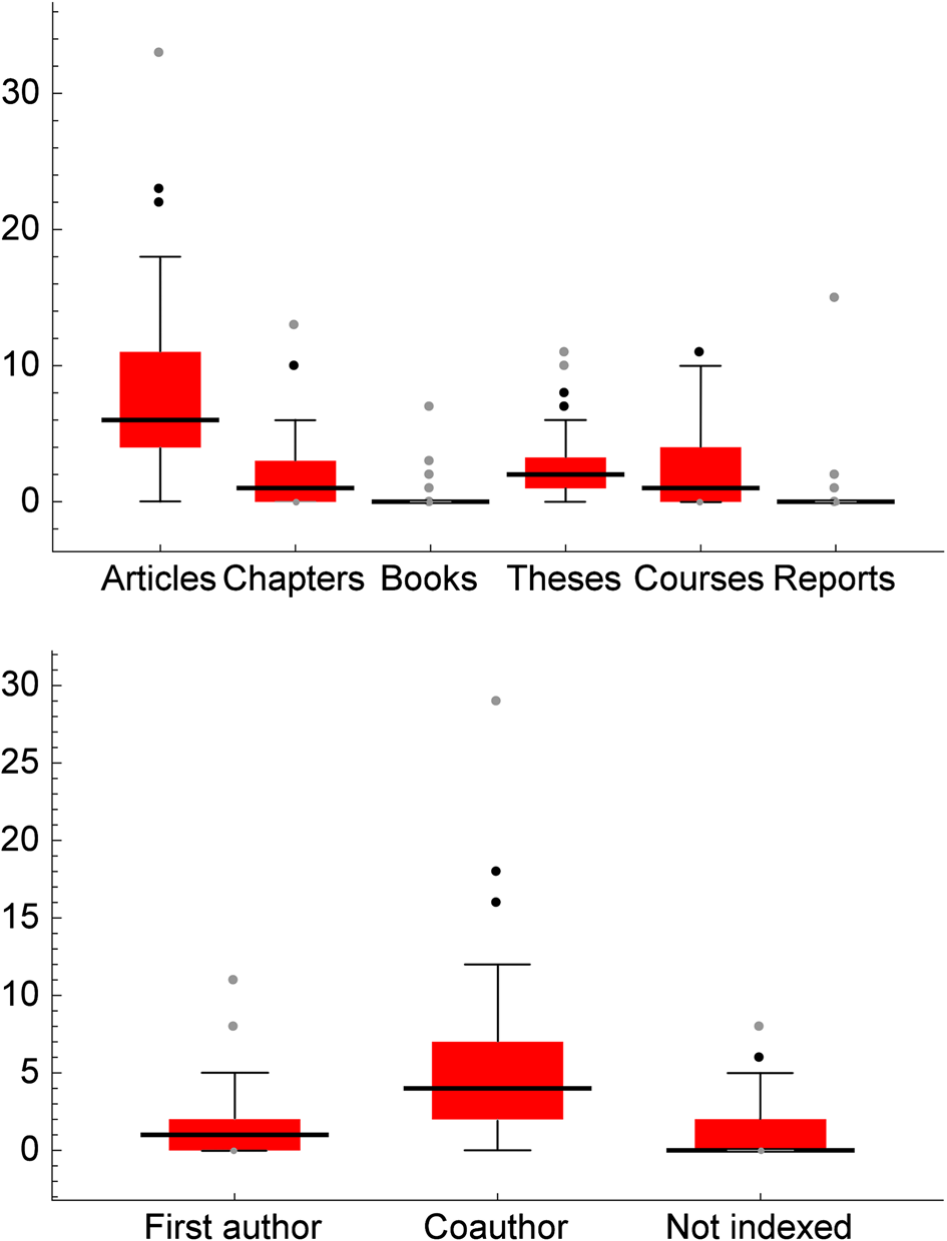
Nine box–whisker plots for the number of products finished at the Biology Institute from 2010 to 2012 by 73 scientists. Medians are given as *horizontal black lines*, interquartile ranges as *boxes*, and overall ranges without outliers as *vertical black lines*. *Black points* are outliers, being at least 1.5-times the interquartile range away from the top of the *box*, and *gray points* indicate far outliers, at least 3-times away. At the *top* the groups are the six major product categories “scientific articles” (indexed, not indexed, first author, coauthor), “book chapters”, “scientific books”, “advisor of theses”, “teacher of courses,” and “technical reports”. The most numerous product is the articles category. At the *bottom*, within the articles category, being coauthor of an indexed article is most frequent, followed by being first author of an indexed article, and finally being author of a non-indexed articles (first author or coauthor)

To get a better appreciation of the data, Fig. 1 (top) shows six box–whisker plots of the major variables of academic output. The first box–whisker plot, on the left in the graph, is for articles, combined as first author or coauthor (indexed or non-indexed). The median number of articles over 3 years was 6 per scientist, i.e., 50 % of the 73 scientists published 6 or less articles, and the other 50 % published 6 or more articles. The interquartile range is from 4 to 11 articles, i.e., 50 % of the scientists are within that range. The range without outliers is from 0 to 18 articles, and there are three outliers (at least 1.5-times the interquartile range away from the top of the box), two of which are considered “far outliers” (at least 3-times away). Comparing the six groups (articles, chapters, books, advising theses, teaching courses, and generating technical reports), two features are notable:

1. The category with the largest median (6) as well as the largest maximum (33) is the one for scientific articles. It is followed by advising theses (median = 2), writing book chapters and teaching courses (1), and writing books or generating technical reports (0). This shows that the emphasis has been placed on publishing scientific articles as the most important activity for a scientist at the institute. Teaching courses is not obligatory at UNAM’s institutes (it is at UNAM’s “facultades”, i.e., “schools”). Over half the scientists did not participate in teaching courses, and did also not generate technical reports.
2. Each product category has a minimum of zero and a maximum of one to several outliers. Given that each scientist presented at least one product, and 50 % of the scientists at least six products, this indicates a high diversity of product mixes of academic output, and raises the issue of how evaluation committees should weigh different products against each other.

Turning in Fig. 1 (bottom) in more detail to the category of articles, one sees that most articles are produced as coauthor, with a median of 4 and an interquartile range from 2 to 7 per scientist over the 3 years. Again, the minimum is 0, and there are outliers as a maximum (notably 29 articles coauthored by one scientist during the 3 years). The median of the articles as first author is 1. Despite the emphasis on publishing in indexed journals, some scientists also continued to publish in journals that are not indexed in Thomson Reuters’s *Journal Citation Reports* (median = 0, maximum = 8).

The PRIDE level and the SNI level are significantly correlated for the 73 scientists, though the relationship is not really linear (not shown here). The problem of poorly behaved residuals in pairwise correlations is the case for many variable pairs from Table 1. For that reason, correlation analysis was not elaborated further.

Though the assignation of an evaluation category in the PRIDE or SNI starts generally at the beginning of the year, the evaluations every few years (generally 3–5) for the PRIDE and SNI are not at the same time for all scientists. Therefore, the data presented here does not necessarily represent exactly the data that the evaluation committees have seen for a given scientist. Nevertheless, it is of interest to present an association between evaluation category in the PRIDE or SNI, with the products presented over the 3-year period.

**Fig. 2:**
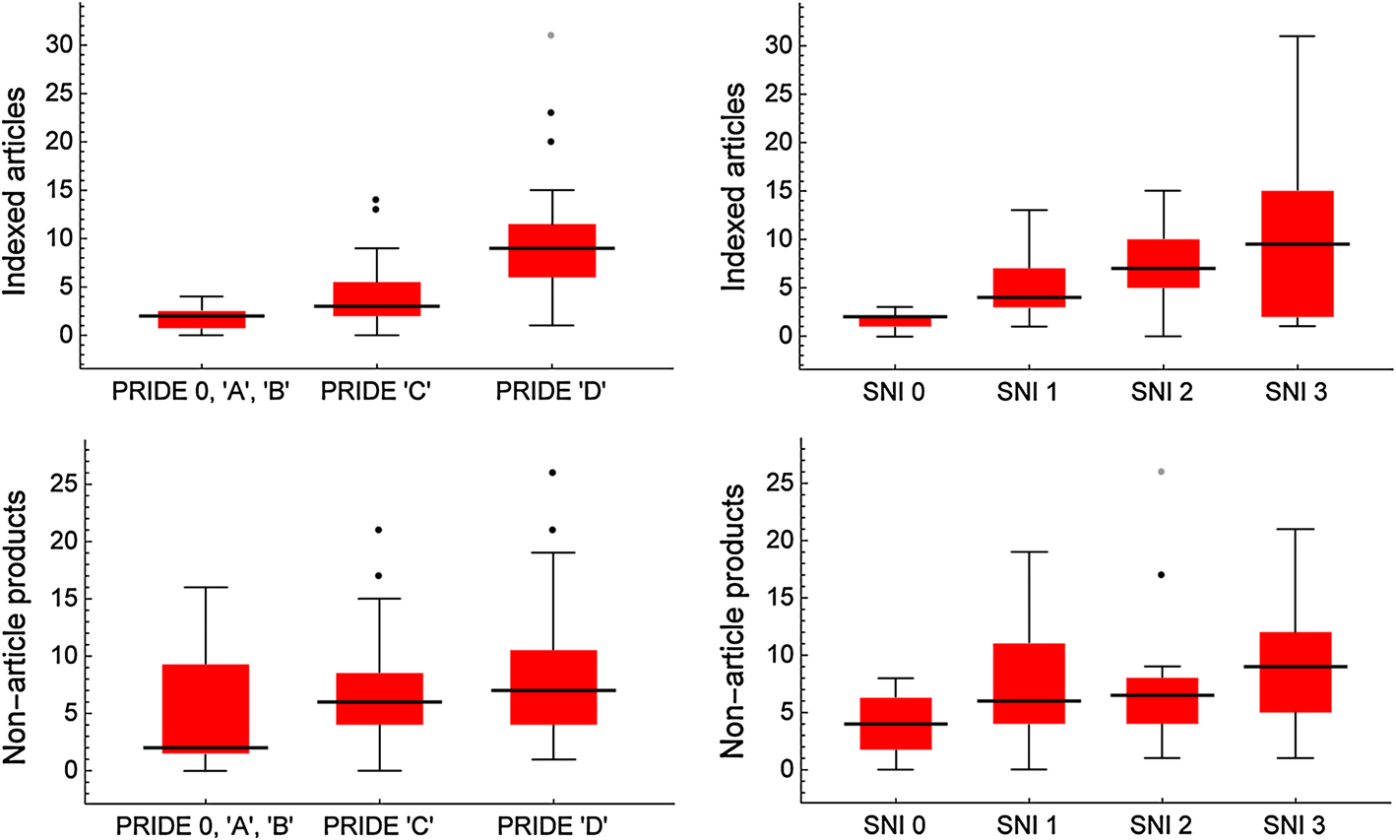
Production of 73 scientists of UNAM’s Biology Institute during 2010–2012: box–whisker plots for the number of articles indexed in the *Journal Citations Reports* (*above*) and the number of all other products or activities (*below*), for UNAM’s reward program PRIDE (*left*) and for Mexico’s nationwide reward program SNI (*right*). Medians are given as *horizontal black lines*, interquartile ranges as *boxes*, and overall ranges without outliers as *vertical black lines*. *Black points* are outliers, being at least 1.5-times the interquartile range away from the top of the *box*, and *gray points* indicate far outliers, at least 3-times away. A level of ‘0’ means that those scientists did not receive rewards from the PRIDE or SNI, respectively. Levels ‘0’, ‘A’, and ‘B’ of the PRIDE were combined because of the low number of scientists in those levels. For statistical significance of pairwise comparisons see Table 2

Figure 2 shows the emphasis that has been placed on indexed scientific articles. The median numbers in the three distinguished levels in the PRIDE, and the four levels in the SNI, increase at higher reward levels. Differences of medians were tested pairwise with the (non-parametric) Mann–Whitney *U* test (Fligner and Policello 1981), as implemented in *Mathematica*. To account for the simultaneous comparisons, the experiment-wide type I error rate *α* was adjusted with the Bonferroni method, dividing it by the number of simultaneous comparisons (Sokal and Rohlf 2012: 239): *α* = $0.05/3$ in case of the PRIDE, and $0.05/6$ in the case of the SNI. Most pairwise comparisons between median differences are highly significant for indexed articles (Fig. 2 top; Table 2). The situation is less obvious when pooling all products, except articles (of any type). Figure 2 (bottom) shows that there is also a tendency to present more products in a higher reward level, but most comparisons are not significantly different (Table 2). The large interval for the first level in the PRIDE is due to the low evaluation of one scientist who presented a high number (15) of technical reports, at the cost of publishing articles instead.

**Table 2:** Pairwise comparisons of medians in Fig. 2, giving the sample sizes (i.e., corresponding number of scientists) and the probabilities from a Mann–Whitney *U* test

	PRIDE ‘C’ (*n* = 36)	PRIDE ‘D’ (*n* = 32)		SNI 1 (*n* = 28)	SNI 2 (*n* = 22)	SNI 3 (*n* = 14)
Number of indexed articles						
PRIDE 0, ‘A’, ‘B’ (*n* = 5)	0.077	**0.0011**	SNI 0 (*n* = 9)	**0.00012**	**0.0010**	**0.0026**
PRIDE ‘C’ (*n* = 36)		**1.9·10** ^−**6**^	SNI 1 (*n* = 28)		**0.033**	0.059
			SNI 2 (*n* = 22)			0.26
Number of non-article products						
PRIDE 0, ‘A’, ‘B’ (*n* = 5)	0.43	0.14	SNI 0 (*n* = 9)	0.061	0.087	**0.016**
PRIDE ‘C’ (*n* = 36)		0.18	SNI 1 (*n* = 28)		0.87	0.21
			SNI 2 (*n* = 22)			0.099

Significant probabilities (*α* = 0.05) are in bold: In those cases the differences between the two medians can be considered significantly different from zero

In conclusion, while the major emphasis by the 73 scientists during 2010–2012 has been on presenting scientific articles (at least as coauthor), there is a lot of variation of presented combinations of products and activities among scientists.

## A numerical algorithm with preference statements

In this section, I propose an algorithm that takes into account trade-offs between product categories, such as not publishing a scientific article while working for example on a book. Priorities to stimulate working on some products should be explicitly made clear to all involved scientists, rather than depend on the personal preferences of the evaluation committees’ members. Furthermore, we would like to avoid surprises, i.e., achieve that the evaluation is largely predictable.

The algorithm would not contemplate anymore the assignation to a discrete level (like level “C” in the PRIDE or level “II” in the SNI), but pay a reward for each recognized product. As a side effect, that would also return the academic prestige back to the stepwise, traditional levels to which a scientist has been promoted (e.g., *Investigador Titular “B”*), instead of depending on the potentially fluctuating levels in the PRIDE or SNI. In other words, in terms of prestige, it would provide more stability, and thus encourage more to work on projects that are of interest, but where the results are less predictable, originate less publications, or take longer to be published.

An appropriate academic evaluation committee could define product categories unambiguously, and subsequently establish relative product values (weights *w*), such as for the 16 product categories for UNAM’s Biology Institute in Table 1. The base value could be the publication of an indexed article, where the to-be-evaluated scientist is first author. The relative values of all other products are then expressed relative to this product. The committee could decide that a non-indexed article with the scientist being first author is worth half an indexed article, that a complete book is worth two indexed articles, and so on (second column in Tables 3, 4). The definition of product categories is essential, but completely free to choose. One may argue, for example, that indexed articles should be subdivided further into “higher-value” indexed articles (in journals of higher prestige in the scientific field) and “lower-value” indexed articles (in journals of lower prestige in the scientific field). Furthermore, scientific books cover a wide range of work and quality, so that the book category should probably be subdivided, e.g., into “short book of national interest”, “extensive book of international interest”, etc. The following approach admits any number of product categories.

**Table 3:** First hypothetical evaluation scenario for 16 product categories and 73 scientists from UNAM’s Biology Institute: balanced values for a product mix

Product category	Relative value (*w*)	Relative price (*p*)	Produced number 2010–2012 (*q*)	Produced value (*p*·*q*)	Share of produced value (*s*) in %	Per-unit US$ value (*V*)
Indexed article (first author)	1	9.2593	109	1,009.2593	17.2	$7,310
Non-indexed article (first author)	0.5	4.6296	31	143.5185	2.4	$3,655
Indexed article (coauthor)	0.5	4.6296	363	1,680.5556	28.7	$3,655
Non-indexed article (coauthor)	0.25	2.3148	57	131.9444	2.3	$1,827
Book chapter (first author)	0.5	4.6296	86	398.1481	6.8	$3,655
Book chapter (coauthor)	0.25	2.3148	58	134.2593	2.3	$1,827
Book (first author)	2	18.5185	9	166.6667	2.8	$14,619
Book (coauthor)	1	9.2593	18	166.6667	2.8	$7,310
Undergraduate thesis advisor	0.5	4.6296	74	342.5926	5.8	$3,655
Master’s thesis advisor	0.75	6.9444	68	472.2222	8.1	$5,482
Doctoral thesis advisor	1	9.2593	29	268.5185	4.6	$7,310
Undergraduate course taught	0.4	3.7037	83	307.4074	5.2	$2,924
Course taught for master’s students	0.6	5.5556	48	266.6667	4.5	$4,386
Course taught for doctoral students	0.8	7.4074	38	281.4815	4.8	$5,848
Technical report (first author)	0.5	4.6296	14	64.8148	1.1	$3,655
Technical report (coauthor)	0.25	2.3148	12	27.7778	0.5	$1,827
Sum	10.8	100	1,097	5,862.5	100.0

**Table 4:** Second hypothetical evaluation scenario for 16 product categories and 73 scientists from UNAM’s Biology Institute: priority on indexed articles

Product category	Relative value (*w*)	Relative price (*p*)	Produced number 2010–2012 (*q*)	Produced value (*p*·*q*)	Share of produced value (*s*) in %	Per-unit US$ value (*V*)
Indexed article (first author)	1	28.5714	109	3,114.2857	20.0	$8,500
Non-indexed article (first author)	0.1	2.8571	31	88.5714	0.6	$850
Indexed article (coauthor)	1	28.5714	363	10,371.4286	66.7	$8,500
Non-indexed article (coauthor)	0.1	2.8571	57	162.8571	1.0	$850
Book chapter (first author)	0.1	2.8571	86	245.7143	1.6	$850
Book chapter (coauthor)	0.1	2.8571	58	165.7143	1.1	$850
Book (first author)	0.1	2.8571	9	25.7143	0.2	$850
Book (coauthor)	0.1	2.8571	18	51.4286	0.3	$850
Undergraduate thesis advisor	0.1	2.8571	74	211.4286	1.4	$850
Master’s thesis advisor	0.2	5.7143	68	388.5714	2.5	$1,700
Doctoral thesis advisor	0.3	8.5714	29	248.5714	1.6	$2,550
Undergraduate course taught	0.1	2.8571	83	237.1429	1.5	$850
Course taught for master’s students	0.1	2.8571	48	137.1429	0.9	$850
Course taught for doctoral students	0.1	2.8571	38	108.5714	0.7	$850
Technical report (first author)	0	0	14	0	0.0	$0
Technical report (coauthor)	0	0	12	0	0.0	$0
Sum	3.5	100	1,097	15,554.3	100.0	

Two fundamentally distinct approaches for evaluating science are the results-oriented and the process-oriented approach (Korhonen et al. 2001: 121; Upton et al. 2014). Here I focus on the results-oriented approach: Which and how many products and activities are reported, and what is their value? In particular for young, beginning scientists, however, a process-oriented approach could be fairer: Have their academic activities been of high quality, even if they have not yet produced publications or other results? In the algorithm proposed here, young scientists could be given a special category of reporting their activities in progress reports.

The sum of all relative values (*w*) in Table 3 being 10.8, and dividing each relative value by this sum, the relative values can be transformed into “relative prices” (third column in Tables 3, 4). The relative prices sum up to 1, but can be multiplied by any factor without affecting relative values; here they are multiplied with the sum of the prices of all product categories being 100, to make the numbers easier to appreciate:

$$ p_{k} = \frac{{w_{k} \cdot 100}}{{\sum\limits_{i = 1}^{n} {w_{i} } }} $$

The index *i* is a counting variable from *i* = 1 to *i* = *n* product categories, while *k* refers to a specific product category among the *n* categories, for which a price or other parameter is calculated. For example for “book chapters (first author)”, the fifth product category in Table 3, *p*_*k*=5_ = $0.5\cdot100/10.8 = 4.6296$. The step of calculating relative prices is not fundamental for the subsequent formulas and discussion, because the information is already included in the relative values; however, the relative prices give a more intuitive notion that one deals with the concept of prices to express values of different products relative to each other, independently of which product serves as the base value.

The fourth column in Tables 3 and 4 gives the number of products from Table 1 (e.g., 109 indexed articles). The per-unit relative prices (*p*) times the quantities (*q*), produced during the evaluation period, is the produced relative value of the work of all 73 scientists together $(p_{ 1} \cdot q_{ 1} + p_{ 2} \cdot q_{ 2} + \cdots )$, being for indexed articles 1,009.2593 in terms of relative prices. The total value for all product categories is 5,862.5. In the next column “Share of produced value,” the produced value is expressed as a percentage of the sum of all produced values:

1 $$ s_{k} = \frac{{p_{k} \cdot q_{k} \cdot 100\;\% }}{{\sum\limits_{i = 1}^{n} {(p_{i} \cdot q_{i} )} }} \, = \frac{{w_{k} \cdot q_{k} \cdot 100\;\% }}{{\sum\limits_{i = 1}^{n} {(w_{i} \cdot q_{i} )} }} $$

For the example of book chapters, *s*_5_ = $$ 4.6296\cdot86\cdot100\;\%/5,862.5 = 6.8\;\%$$. Next, the absolute per-unit US$ value (*V*) in the last column of Tables 3 and 4 is calculated as:

2 $$ V_{k} = \frac{{p_{k} \cdot T}}{{\sum\limits_{i = 1}^{n} {(p_{i} \cdot q_{i} )} }} \, = \frac{{w_{k} \cdot T}}{{\sum\limits_{i = 1}^{n} {(w_{i} \cdot q_{i} )} }} $$

The variable *T* is the total reward money for all scientists in the evaluation system (here paid by the university). This is the key formula for calculating the rewards. The formula results from converting the created share of relative value in a given product category into a share of absolute value, $$ p_{k} \cdot q_{k} /\sum_{i = 1}^{n} (p_{i} \cdot q_{i} ) = V_{k} \cdot q_{k} /T $$, and solving for *V*_*k*_. In 2012, the total amount of money available for the PRIDE rewards in the Biology Institute was 38,567,651 Mexican pesos, which—at an exchange rate of 12.5 pesos per dollar—translates into US$3,085,412. Estimating that half of this amount is for the 73 scientists (and the other half for academic assistants), and multiplying by three years, results in *T* = US$4,628,118 as the PRIDE reward money for the years 2010–2012 together. Applying Eq. 2 to the category of “book chapters (first author),” one gets *V*_5_ = 4.6296·US$$$4,628,118/5,862.5$$ = US$3,655 for each book chapter.

Given the (hypothetical) relative values of the scenario of Table 3, the highest reward with US$14,619 is for publishing a scientific book as first author. The lowest reward with US$1,827 is equally for finishing as coauthor a non-indexed article, a book chapter, or a technical report. This first scenario is called “balanced values for a product mix,” because the median relative value (*w*) is 0.5 (the mean 0.68), and the interquartile range from 0.45 to 0.9. Furthermore, there is no product with a zero-value, relative to the base value of 1 for an indexed articles as first author.

The second scenario is called “priority on indexed articles”, with a median relative value for all product categories of 0.1 (mean of 0.22), the interquartile range from 0.1 to 0.15, and technical reports receiving a relative value of 0. As a result, the reward is in a range from US$0 for technical reports to US8,500 for indexed articles (as author or coauthor). Technical reports from collaboration with industry or other entities of society would be completely discouraged by the PRIDE. The total produced value with 15,554.3 relative units is 2.7-times higher than in the first scenario (5,862.5), because the relative values are higher for products that are produced in higher quantity.

**Fig. 3:**
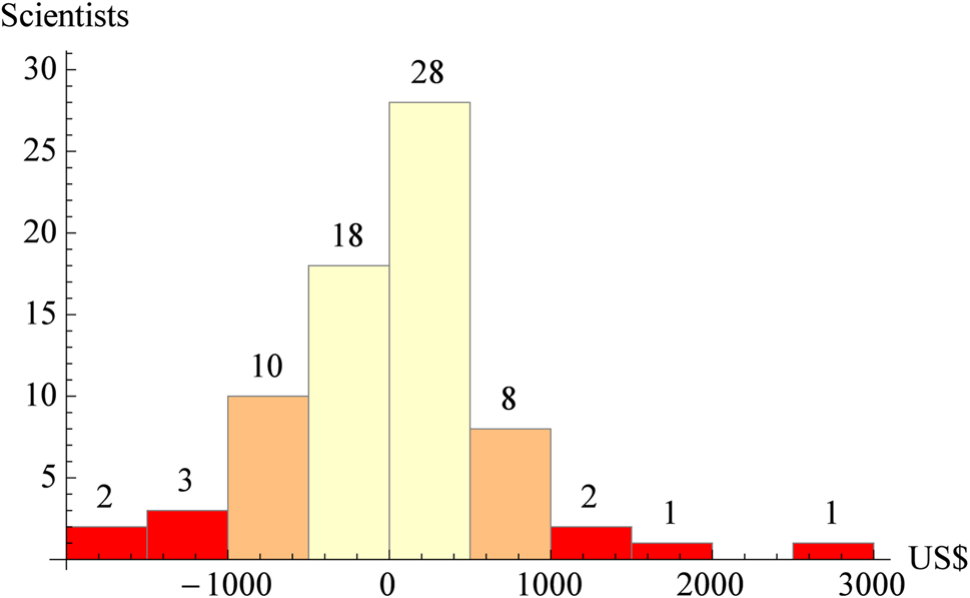
Histogram of the reward differences between two hypothetical evaluation scenarios for the 73 scientists of UNAM’s Biology Institute, when the relative values of the first scenario should be used (Table 3), but the relative values of the second scenario are applied (Table 4). In that case, some scientists would get underpaid by up to almost US$2,000 per month, while others would get overpaid by up to almost US$3,000. The incorrect reward assignment of (here) 29 % of the program’s budget would tend to distort academic priorities of the scientists

What is the effect of applying one versus the other scenario to the 73 scientists of UNAM’s Biology Institute? The Biology Institute belongs to a public university, being paid for overwhelmingly by the Mexican federal government in benefit of society. Let us assume that the majority of Mexico’s society prefers the relative values of the first scenario (Table 3), but the institute uses the second scenario for its reward program (Table 4). The UNAM pays approximately US$128,559 per month $$(4,628,118/36)$$ for the Biology Institute’s PRIDE, on average US$1,761 for each of the 73 scientists, independently of the applied scenario. For each scientist we can calculate the monthly reward over the next 3 years, according to their performance over the last 3 years, when applying either one of the scenarios in Tables 3 and 4. For the first scenario, the median monthly reward would be US$1,574, in a range from US$102 to US$4,802. For the second scenario, the median would be US$1,535, in a range from US$24 to US$7,744. Subsequently the difference between the two scenarios for each scientist can be calculated. Figure 3 shows a histogram of those differences. There are 33 scientists who would be underpaid, in a range from US$2 to $1,697 per month (negative values). On the other hand, there are 40 scientists who would be overpaid, in a range from US$2 to $2,942 per month (positive values). Since the total amount *T* is fixed, the amount of underpayment is equal to the amount of overpayment, and both are US$18,376 per month. Consequently, $$29\;\%\,(18,376\cdot2\cdot100\;\%/128,559)$$ of the monthly reward budget would be wrongly assigned. Paying almost a third of the reward money incorrectly obviously would tend to distort academic priorities. Consequently, determining preferences is a crucial step that needs to be done explicitly in an academic institution.

It is convenient to calculate a sensitivity analysis, to see how sensitive the per-unit rewards in US$ are to the relative values, i.e., to the decisions of the evaluation committee, as well as to the produced quantities. Since deriving and exemplifying the corresponding formulas is a rather technical issue, the details of the sensitivity analysis are presented in the Appendix. The general conclusions from the sensitivity analysis, however, are the following:

1. A percentage change of the relative value for a given product category (*w*_*k*_) will always cause a percentage change of its per-unit US$ value (*V*_*k*_) that is smaller. The same is true for a percentage change of the produced quantity in a given product category (*q*_*k*_).
2. When increasing the relative value for a given product category (*w*_*k*_), its per-unit US$ value (*V*_*k*_) goes asymptotically towards a maximum percentage change (here for a published book chapter it is 1,372 %).
3. With increasing production number in a given product category (*q*_*k*_), its per-unit US$ value (*V*_*k*_) is going asymptotically towards zero.
4. If the quantities in all product categories are changed by the same factor, the per-unit US$ value in each product category changes by the inverse of that factor.
5. A 10 %-change of the quantity in a given product category (*q*_*k*_) has in general less impact on its per-unit US$ value (*V*_*k*_) than a 10 %-change of its relative value (*w*_*k*_). This is relevant in that the emphasis on the relative value in the product categories is generally more important than fluctuations of the production numbers.
6. Finally, a change of the total reward amount (*T*) by a certain factor simply increases each per-unit US$ value proportionally by this factor.

## The algorithm in summary

In this section the approach and algorithm is presented in an integrated way. First, an evaluation committee would have to go through the following steps:

1. Make clear whose values are supposed to be reflected, when putting a value on a scientist’s performance. On the one hand, we could think of basic science, where the values of the most advanced peer scientists in the field should be reflected. On the other hand, we could think of applied science, where the values of actual or potential users and the educated laymen in society should be reflected.
2. Make a list of all acceptable products to consider: Which scientific products and activities are of interest to the institution, in addition to scientific articles, and thus are to be included in the evaluation: Publication of scientific books? Teaching? Collaboration with industry, governmental sectors, etc.? Curation of scientific collections? Publication of scientific information in non-scientific journals? Institutional development? Patents?
3. Each product category needs a definition and a circumscription of what minimum characteristics make the product or activity acceptable in the evaluation. For example, a 10-page small, stand-alone flora volume should not be evaluated as a book, but could be evaluated as a book chapter. Which journals are acceptable for the publication of scientific articles? Should the scientific-article category or book category be subdivided into higher-value and lower-value products? What are the characteristics of an acceptable technical report? Conceptually, the reward is given under a threshold criterion, i.e., a per-unit minimum quality has been achieved. The product is not judged individually further!
4. Define minimum requirements for scientists to be able to enter the reward program. Examples are publishing as first author at least one indexed scientific article, ${\text{and}}/{\text{or}}$ presenting a minimum of six finished products (or activities) over a 3-year period.
5. In order to avoid trading excessively quality for quantity, define maximum numbers to be accepted in each product category. If a maximum is defined and a scientist elaborated a higher number of products in that category, the scientist can choose which ones to present.
6. Define which product category shall have the base value 1. An obvious choice is the category of “indexed articles (first author)”. Then define the value of each other product category relative to the one with the base value. This determines the column “Relative value (*w*)” in Tables 3 and 4.

The input for the algorithm consists of the following information:

- The product categories of Point 3 above, as given in the first column in Tables 3 and 4.
- The assigned relative values (*w*) of Point 6, as in the second column in Tables 3 and 4.
- For each to-be-evaluated scientist the number of products generated during the evaluation period. This information is not shown here, because it would be a matrix of the 16 product categories times the 73 scientists. The sum for each product category for all 73 scientists together, however, is again shown, in the fourth column in Tables 3 and 4 as “Produced number 2010–2012 (*q*)”. While the to-be-evaluated scientists should assign their products to the different categories, these assignments need to be reviewed by the evaluation committee, to avoid and possibly correct wrong assignments of products to the categories. This process in turn provides feedback to the evaluation committee to see if the product category definitions work adequately or need to be modified.
- The assigned budget for all scientists together, corresponding to the evaluation period (total reward money *T*).

The subsequent algorithm can be implemented for example in an Excel spreadsheet, in order to carry out the following sequence of step:

(I) Calculate the produced value (*p*·*q*) for each product category and the sum for all product categories, as given in the fifth column of Tables 3 and 4.
(II) Calculate the per-unit value (*V*) in monetary currency (here US$) with Eq. 2, as given in the last column of Tables 3 and 4.
(III) For each scientist, multiply the per-unit value with the number of submitted products in each category (possibly only up to a defined maximum). This is the total amount to be awarded for the (here) 3-year evaluation period.
(IV) Calculate as additional (optional) information the relative prices (*p*) and shares of produced value (*s*), as given in the third and sixth column in Tables 3 and 4.
(V) Also as additional information, calculate the sensitivity analysis with Eqs. 4 and 5 in the Appendix.

The output of the algorithm is the amount of money to be awarded to each scientist of the institution for the achievements during the evaluation period. In case of the PRIDE or SNI, the amount for each scientist would be divided by the 36 months to get a monthly reward to be paid during the next evaluation period. Furthermore, the additional information from Points IV and V should be presented and analyzed by the evaluation committee, to guide possible adjustments. Finally, the evaluation committee would need to specify periodically what changes it makes to the definitions of the product categories and the relative values (*w*) for each product.

## Discussion

At the heart of science is the implementation of a creative idea of scientific interest. According to Popper (1959: 27), “a scientist, whether theorist or experimenter, puts forward statements, or systems of statements, and tests them step by step. In the field of the empirical sciences, more particularly, [s]he constructs hypotheses, or systems of theories, and tests them against experience by observation and experiment”. Academic work includes additional activities, such as teaching, scientific extension, and technical services. Korhonen et al. (2001: 123) define as ideal “a research unit whose members continuously produce high quality, innovative and internationally recognized research, and who actively supervise doctoral students and actively take part in various activities of the scientific community”. Evaluation of scientific—or more broadly academic—performance is a fundamental aspect at scientific institutions, in order to distinguish and reward scientists with both money and prestige.

The classical form of a scientific evaluation consists of a stepwise process throughout a career, where a candidate has to convince a committee of senior peers that a promotion to a higher employment category is justified, a category from which he or she cannot fall back again to a lower one. The underlying idea is that scientists build a scientific legacy throughout their career. Such a legacy typically consists of new knowledge, better prepared human resources, and innovative applications, available for society’s next generation. The necessary number of publications may vary greatly with the scientific field, the scientist’s focus, the innovative steps, etc. Ultimately, scientific publications are one important medium to build such a legacy, but nevertheless they are only a medium and not the goal itself. The number of published articles or the number of received citations do not represent necessarily an indicator of the achieved legacy. Furthermore, trading-in quality and innovation for quantity, and restricting the notion of productivity to maximizing the number of scientific articles, is counterproductive to building an academic legacy.

The promotional steps are obviously crucial in the classical system, which generally is still implemented at universities. García-Aracil et al. (2006) conclude in their study of the research evaluation system in (the autonomous community of) Valencia in Spain that the peer review process is not as objective as they expected. This coincides with the critical comment from John Bailar (Cicchetti 1991: 137) that “the purpose of peer review is not reliability [of achieving the same evaluation among peers], but to improve decisions concerning publication and funding”. Reliability and predictability, however, are generally considered important objectives for periodic career evaluations every few years, like in the PRIDE and SNI. The approach proposed here provides one way to structure the peer-review process for career evaluations such as to make the outcome largely predictable. Consequently, the presented approach does not represent peer review in the traditional way, where an evaluator looks through the to-be-evaluated products of a scientist and a list of evaluation criteria, in order to provide a—generally subjective—opinion of the combined value of these products. Rather, the members of the evaluation committee are peer reviewers in the sense that they understand in a given institution what are valid product categories, how these categories should be defined exactly, how much work is involved in elaborating products in a given category, what relative value is appropriate given that work and institutional objectives, and being able to review if submitted products coincide with the defined categories.

Evaluation is “the act of estimating the value or worth,” and value in our context refers to “a fair or proper equivalent in money” (McKechnie 1983: 2018). If values are defined in terms of money, values translate into prices (Pearce 1995: 446). The other way round, prices represent the values that people put on goods and services. A problem of evaluating scientists is that many products and services are public goods that benefit society, but not a private investor, and consequently there are no market prices to assess their worth. For that reason, academic evaluation committees, instead of markets, are generally in charge of evaluating their peers (colleagues). Each committee member needs to make a value judgement. If we follow Popper (1959: 44, citing Immanuel Kant) that “subjectivity refers to feelings of conviction”, then such value judgements are intrinsically subjective. There is no way to get around that subjective judgement in evaluations, and thus there is no stand-alone objective algorithm to evaluate the performance of scientists, i.e., a computer cannot by itself carry out completely the evaluation. Once preference statements are made by humans, however, an algorithm can work through the details of assigning rewards, and be objective in the sense that it can be tested and understood by anybody.

It is remarkable that the role of preferences for the evaluation of scientific performance is so little discussed or explicitly stated. Roessner (2000: 128) formulates the question differently as “Where do I want to go?” The lack of explicit preference indications probably has to do with the notion of “academic freedom”, but at the latest during decisions about funding and promotions the influence of evaluators’ preferences becomes unavoidable. While equal treatment of the development of all areas of knowledge by independently working scientists is considered an academic ideal, in practice the funding by governments or industry, and its distribution by academic committees, imposes quite different values for different research areas or academic activities. By not stating preferences explicitly and concretely, or at least not sufficiently, as has occurred in the PRIDE or SNI programs, “pseudo-objective” evaluation results are generated, without the necessary institutional discussion about “where to go”. The question is not only about the priorities among different possible academic products and services, but also in a stricter scientific sense about what type of research should be supported. Korhonen et al. (2001: 122) and Nagpaul and Roy (2003) recognize the problem of choosing among multiple objectives in academic work, but their more complicated algorithms take preferences in a more indirect way into account.

Scientists are major drivers of “pure utility growth” (Ricker 1997). Pure utility growth occurs when in general a better or more appreciated good or service is produced, while prices remain the same, i.e., you get a better product for the same price. Better computers and medical advances are obvious examples. Pure utility growth is obviously of great interest as a driver of human development. Taking adequately creative and innovative contributions of scientific work into account is one of the most intangible aspects of evaluation, for both a computer algorithm and an evaluation committee. An operational definition of “creativity” is given by Sternberg et al. (2005): “Creativity is the ability to produce work that is novel (i.e., original, unexpected), high in quality, and appropriate (i.e., useful, meets task constraints).” When is a contribution truly novel? How can one measure its quality? For whom or what is it appropriate? Some ideas to get answers for evaluation purposes are the following:

- A high priority on quality, frequently at the expense of output quantity, is fundamental for scientific achievement. Consequently, it seems wise to define a maximum number of products to be considered for reward assignment, in order to focus the reward on creativity and quality, rather than on maximizing the number of academic products. For example, the limit could be three times the required minimum number, such as three indexed articles as first author, during a 3-year period, ${\text{and}}/{\text{or}}$ a total of 18 products or activities. Scientists are of course free to produce the number of articles, etc., they want and are able to. If they have more products than the limit admits, they could choose their best ones for evaluation. While at a first glance the restriction might appear intrusive, it would cause the system to constitute a mixture between a traditional system of complete freedom (depending on the position) and a performance-based system that tends to prioritize (excessively) quantitative results. While academia has to show concrete and in some form measurable results as a function of funding, its essence is to generate difficult-to-measure products and services, such as new knowledge, preparing students, providing scientific advice, or contributing to culture. The primary objective of a scientist should not be to produce a lot of (printed or electronic) text, but to produce and implement a lot of new knowledge. That objective should not be distorted by offering the wrong rewards.
- With a median of four coauthorships during 3 years (i.e., not being first author), many scientists of the Biology Institute based much of their performance on coauthoring articles (Table 1; Fig. 1 bottom). If coauthorship translates into money, then coauthorship becomes easily one of the most abused concepts for evaluation. A coauthor can be anybody from a person who actually wrote the whole article, but honors another person as first author, to somebody who did not even read the article, but is for example responsible for the laboratory where the first author worked. Some journals, such as *PLOS ONE*, state for each coauthor what contribution he or she made to the article, including who conceived the idea, who performed the experiments, who analyzed the data, and who contributed to the writing of the article. For evaluation purposes, it would be important to implement this requirement for journals in general, and to consider the type of contribution of the coauthor.
- While one can discuss any convention and arrangement, I would question the idea to include any number of people as coauthors in a scientific article, where traditionally the first (lead) author is supposed to have made a creative contribution and any coauthor is supposed to have had a direct and usually intellectual participation in the development of the manuscript. An example that is contrary to this idea is a physics article by Atlas Collaboration (2012), where over 3,000 coauthors from 178 institutions are listed at the end of the article. Most of those “coauthors” should be in the acknowledgement, if they need to be mentioned at all. At least for the evaluation of scientific performance of the authors, such an article seems inappropriate. One may argue that any number of coauthors over ten should include a justification (e.g., that different coauthors developed different sections of the manuscript), and does not represent inflated coauthorship.
- The to-be-evaluated scientist could present a statement, explaining novelty, quality, and usefulness. If convincing as especially creative, the relative value of the corresponding product could be augmented. For example, an indexed article as first author could be given in that case the relative value of 2 (“highly creative article”) instead of 1. Also, for long-term projects that surpass the evaluation period, a progress report could be considered for evaluation.
- As stated by Nicolaisen (2007: 609), one application of citation analysis is the “qualitative and quantitative evaluation of scientists, publications, and scientific institutions”. In basic research, citation analysis can potentially be of some interest. There are, however, some conventions that obscure citation analysis for evaluation purposes. First, there is no distinction between *fundamental citations* and *substitutable citations*. Many citations are just side-notes in an article, stating that somebody else has also worked on the topic (or a related one); those citations can easily be substituted or even completely omitted. Relatively few citations are of such importance that the idea of the previous article enters truly into the new text, that one has learnt from it, or that the new article could not even have been written without the advances from the cited article. An example is a new method that is applied and cited in a subsequent article. Second, citations to own papers are generally excluded. Auto-citations are, however, frequently fundamental citations, because one follows up on previously developed ideas or methods, a process that should be rewarded. Ultimately, each article’s author(s) would need to indicate in the article which references are fundamental (all others being substitutable). For example, in the present article I would consider the following references fundamental: Batey (2012), Cicchetti (1991), Korhonen et al. (2001), McKechnie (1983), Pearce (1995), Popper (1959), Ricker (1997), Ricker et al. (2009), and Sternberg et al. (2005). Consequently, only 9 (37.5 %) of my 24 references I would consider “fundamental” or “central” to this article, i.e., a little over a third.
- For applied research in the sense of “solutions-for-society research”, or research for or by private companies (Hauser and Zettelmeyer 1997), citation analysis is inappropriate, because non-publishing users will not cite the research, even though it may have a tremendous impact (just think of research for developing and improving computers). Funding agencies and research-policy politicians largely like to focus on that type of research. To discourage it by the inappropriate use of citation analysis is counterproductive for funding science. A statement by the authors and representatives of interested users about the impact of the research could provide a more appropriate basis for evaluation, at least to clearly determine whether it does or does not represent useful applied research.

The limitations of numerical algorithms, mentioned in the introduction, are largely addressed by the approach proposed here: Preferences are explicitly taken into account, creativity could be distinguished to some extent, the evaluation is highly flexible to consider multiple product categories, and a maximum limit of taken-into-account products would avoid an excessive focus on quantity.

There is also the issue of institutional diversity that is important in science. While programs like the PRIDE and especially the SNI for whole Mexico has stimulated increased production in indexed journals under international-quality criteria (Luna-Morales 2012), the side effect has been the uniformization of the evaluation criteria at UNAM (PRIDE) and even in all Mexican research institutions (SNI). The priority on generating articles indexed by Thomson Reuters (Figs. 1, 2) has also been largely uniformized for all scientific fields, as diverse as for example astronomy and agricultural sciences. That is undesirable, because depending on the scientific field and position of an institution in a range from completely basic to completely applied research, the specialization of institutions on different academic product mixes is efficient and important for society.

## Appendix: Sensitivity analysis

Since an evaluation committee would decide about relative values (or weights *w*) and not relative prices (*p*), in the following equations only relative values are considered. If *c*_*w*_ is a percentage of *w*_*k*_ for the *k*’s product category, to be added to *w*_*k*_ (*c*_*w*_ can also be negative), we get the formula for the new value of *V*_*k*_, calculated from the original parameters:

3 $$ V_{{k,c_{w} }} = \frac{{\left( {1 + \frac{{c_{w} }}{100\;\% }} \right) \cdot w_{k} \cdot T}}{{\sum\limits_{i = 1}^{n} {(w_{i} \cdot q_{i} )} + \frac{{w_{k} \cdot q_{k} \cdot c_{w} }}{100\;\% }}} $$

The formula is derived from Eq. 2, multiplying *w*_*k*_ with $(1 + {c}_{w}/100\;\%)$. The idea is to leave the term $\sum _{i = 1}^{{^{n} }} (w_{i} \cdot q_{i} )$ intact, because that simplifies calculations for each product category by computing the term only once. Therefore the denominator is calculated as $\sum\nolimits_{i = 1}^{n} {(w_{i} \cdot q_{i} )} - w_{k} \cdot q_{k} + w_{k} \cdot \left( {1 + \frac{{c_{w} }}{100\;\% }} \right) \cdot q_{k} = \sum\nolimits_{i = 1}^{n} {(w_{i} \cdot q_{i} ) + w_{k} \cdot q_{k} \cdot c_{w} /100\;\% }$. For applying Eq. 3, we need $\sum _{i = 1}^{n} (w_{i} \cdot q_{i} )$, which was not calculated in Tables 3 or 4. However, because of Eq. 1, we have the following relationship:

$$ \sum\limits_{i = 1}^{n} {(w_{i} \cdot q_{i} )} = \frac{{\left( {\sum\limits_{i = 1}^{n} {w_{i} } } \right) \cdot \sum\limits_{i = 1}^{n} {(p_{i} \cdot q_{i} )} }}{{\sum\limits_{i = 1}^{n} {p_{i} } }} $$

Therefore, we can calculate $$ \sum _{i = 1}^{n} (w_{i} \cdot q_{i} ) $$ quickly from Table 3 as $$ 10.8\cdot5,862.5/100 = 633.15$$. Let us consider a 10 % change of the relative value of a book chapter for the first author, from 0.5 to 0.55 units (of the value of an indexed article). Then *c*_*w*_ = 10 % and $$ V_{{k,c_{w} }} $$ = 1.1·0.5·US$$$ 4,628,118/[633.15\;+\;0.1\cdot0.5\cdot86]$$ = US$3,993. We are, however, more interested in its relative change in percent, i.e., $$ {{\left( {V_{{k,c_{w} }} - V_{k} } \right) \cdot 100\;\% } \mathord{\left/ {\vphantom {{\left( {V_{{k,c_{w} }} - V_{k} } \right) \cdot 100\;\% } {V_{k} }}} \right. \kern-0pt} {V_{k} }} $$. With Eqs. 2 and 3 we get:

4 $$ {RCV}_{{k,c_{w} }} = \frac{{c_{w} \cdot \left( {\sum\limits_{i = 1}^{n} {(w_{i} \cdot q_{i} ) - w_{k} \cdot q_{k} } } \right)}}{{\sum\limits_{i = 1}^{n} {(w_{i} \cdot q_{i} )} + \frac{{w_{k} \cdot q_{k} \cdot c_{w} }}{100\;\% }}} $$

Thus, the 10 % increase of the relative value leads to a $$10\;\%\cdot(633.15\;-\;0.5\cdot86)/(633.15\;+\;10\;\%\cdot0.5\cdot86/100\;\%) = 9.3\;\%$$ increase of the absolute per-unit US$ value *V*_*k*_. Applying Eq. 4 to each product category, an increase of 10 % in the relative value (*w*) results in an increase of the per-unit US$ value (*V*) in a range from 6.9 to 9.9 %, depending on the product category. A decrease by 10 % results in a decrease in the range from 7.3 to 9.96 %. A percentage change of *c*_*w*_ of the relative values will always cause a percentage change of the per-unit values that is smaller than *c*_*w*_. This can be shown by setting Eq. 4 for $$ {RCV}_{{k,c_{w} }} < c_{w} $$, which results in *c*_*w*_ > −100 %. This is always true, because the relative values have to be positive to make sense.

Similarly to analyzing a change of the relative value (*w*), one can analyze a change of the quantity (*q*) produced in the institution. The formula for the per-unit US$ value $$ V_{{k,c_{q} }} $$ that results from changing *q*_*k*_ by a percentage *c*_*q*_ is:

$$ V_{{k,c_{q} }} = \frac{{w_{k} \cdot T}}{{\sum\limits_{i = 1}^{n} {(w_{i} \cdot q_{i} )} + \frac{{w_{k} \cdot q_{k} \cdot c_{q} }}{100\;\% }}} $$

For the example of increasing the number of published book chapters over 3 years in the Biology Institute by 10 % from 86 to 94.6, $$ V_{{k,c_{q} }} $$ = 0.5·US$$$ 4,628,118/[633.15\;+\;0.5\cdot86\cdot10\;\%\;/\;100\;\%]$$ = US$3,630. The 10 % number is kept for comparison, even if the resulting number of book chapters becomes non-integer (for 95 chapters, the change would be 10.5 %). Note that an increase of quantity leads to a decrease of the absolute value, because the total amount of money for all products is fixed. The corresponding relative change (in percent) is:

5 $$ {RCV}_{{k,c_{q} }} = \frac{{ - w_{k} \cdot q_{k} \cdot c_{q} }}{{\sum\limits_{i = 1}^{n} {(w_{i} \cdot q_{i} )} + \frac{{w_{k} \cdot q_{k} \cdot c_{q} }}{100\;\% }}} $$

In our example, changing *q*_*k*_ by *c*_*q*_ = 10 %, $$ {RCV}_{{k,c_{q} }} = - 10\;\% \cdot0. 5\cdot 8 6/\left( { 6 3 3. 1 5 + 10\;\% \cdot0. 5\cdot 8 6/ 100\;\% } \right) = - 0. 6 7\;\% $$. Applying Eq. 5 to each product category, an increase of 10 % in the quantity results in a decrease of the per-unit US$ value in a range from 0.05 to 2.8 %, depending on the product category. A decrease by 10 % results in an increase in the range from 0.05 to 2.95 %. Referring to an absolute (or modulus) value with *Abs*, one can show that the absolute value of $$ {{RCV}}_{{k,c_{q} }} $$ will always be smaller than the absolute value of *c*_*q*_, i.e., that $$ V_{{k,c_{q} }} $$ is not overly sensitive to *c*_*q*_: given positive *w*_*k*_ and *q*_*k*_, simplifying

$$ {Abs}\left[ { - w_{k} \cdot q_{k} \cdot c_{q} / \left( {\sum\limits_{i = 1}^{n} {(w_{i} \cdot q_{i} ) + w_{k} \cdot q_{k} \cdot c_{q} / 100\% \;} } \right)} \right] > Abs \left[ {c_{q} } \right]{\text{ leads to}}\; Abs \left[ {c_{q} } \right] > 0, $$

which is always true.

The relative change *RCV*_*k*_ as a function of *c*_*w*_ (upward sloped and right-winged) or of *c*_*q*_ (downward sloped and left-winged), respectively, is asymptotical. For *c*_*w*_ going towards infinity, $$ {RCV}_{{k,c_{w} \to \infty }} = {{100\;\% \cdot \left( {\sum\nolimits_{i = 1}^{n} {(w_{i} \cdot q_{i} ) - w_{k} \cdot q_{k} } } \right)} \mathord{\left/ {\vphantom {{100\;\% \cdot \left( {\sum\nolimits_{i = 1}^{n} {(w_{i} \cdot q_{i} ) - w_{k} \cdot q_{k} } } \right)} {w_{k} \cdot q_{k} }}} \right. \kern-0pt} {w_{k} \cdot q_{k} }} $$. The formula is derived by dividing denominator and numerator in Eq. 4 by *c*_*w*_. The only term containing *c*_*w*_ is subsequently $$ \sum_{i = 1}^{n}  (w_{i} \cdot q_{i} )/ c_{w} $$, which for *c*_*w*→∞_ goes to zero. One sees that the maximum relative change is the originally produced value of the products other than book chapters, as a percentage of the originally produced value of book chapters. For our example, this maximal change of the US$ value of a published book chapter is $$ 100\;\%\cdot(633.15 - 0.5\cdot86)/(0.5\cdot86) = 1,372\;\%$$.

For *c*_*q*_, the asymptote is $$ {RCV}_{{k,c_{q} \to \infty }} = - 100\;\% $$, meaning that with increased production in product category *k*, the per-unit US$ value $$ V_{{k,c_{q} }} $$ is decreasing towards zero. If the quantities in all product categories are changed by the same factor *f*, the per-unit US$ value of each product category changes by the inverse of that factor, because Eq. 2 converts to $$ w_{k} \cdot T/\sum _{i = 1}^{n} (w_{i} \cdot f \cdot q_{i} ) = V_{k} /f $$.

A 10 %-change of the quantity in a given product category (*q*_*k*_) has in general less impact than a 10 %-change of its relative value (*w*_*k*_). This is relevant in that the emphasis on the relative values (i.e., relative prices) in the product categories is generally more important than fluctuations of the production numbers. One can see this by dividing Eqs. 4 by 5, when *c*_*w*_ = *c*_*q*_ = *c*:

6 $$ \frac{{{RCV}_{{k,c_{w} }} \;{\text{with }}c_{w} = c}}{{{RCV}_{{k,c_{q} }} \;{\text{with }}c_{q} = c}} = \frac{{ - \sum\limits_{i = 1}^{n} {(w_{i} \cdot q_{i} )} - w_{k} \cdot q_{k} }}{{w_{k} \cdot q_{k} }} $$

In our example of book chapters, the ratio from Eq. 6 is $-(633.15\;-\;0.5\cdot86)/(0.5\cdot86)\;=\;-13.72$. A percentage change of the relative value (*w*_*k*_) will have a larger relative impact on the product reward than the same percentage change of the produced quantity (*q*_*k*_), if the absolute value of the ratio given with Eq. 6 (here 13.72) is greater than 1. Since *w*_*k*_·*q*_*k*_ forms part of the term $\sum_{i = 1}^{n}  (w_{i} \cdot q_{i} )$, this is the case if the combined relative value in all product categories other than *k* is greater than the relative produced value in product category *k*. This will generally be true.

Finally, a change of the total reward amount *T* by a factor *f* simply increases each per-unit reward *V*_*k*_ by the same factor, i.e., *V*_*k*,*f*_ = *V*_*k*_·*f*. This can be seen from Eq. 2.
